# Academic climate and psychopathological symptomatology in Spanish medical students

**DOI:** 10.1186/s12909-023-04811-2

**Published:** 2023-11-07

**Authors:** Montse Esquerda, Joaquín Garcia-Estañ, Albert Ruiz-Rosales, J. Miguel Garcia-Abajo, Jesus Millan

**Affiliations:** 1 Sociedad Española de Educación Médica (SEDEM), Madrid, Spain; 2 Institut Borja de Bioética, Universitat Ramon Llull, Barcelona, Spain; 3 Facultad de Medicina, Universitat de Lleida, Lleida, Spain; 4 Center of Studies On Medical Education, University of Murcia, Murcia, Spain; 5 Consejo Español de Estudiantes de Medicina (CEEM), Madrid, Spain; 6 Medical Student at University of Barcelona, Barcelona, Spain; 7 Medical Student at the Autonomous University of Madrid, Madrid, Spain; 8 Universidad Complutense de Madrid, Madrid, Spain

**Keywords:** Academic climate, Learning Environment, Medical students, Mental health, Psychological Distress

## Abstract

**Introduction:**

Medical Education studies suggest that medical students experience depression, anxiety and psychopathological symptomatology in a proportion higher than in the rest of the population. In the present study, we aimed to conduct a nationwide analysis to describe student’s perceptions of Educational Climate in Spanish medical schools, and its relationship with psychopathological symptomatology.

**Methods:**

The study was carried out in 2022 in all 44 medical schools in Spain, and analyses the academic climate, and psychopathological symptomatology among medical students (*n* = 4374). To measure these variables, we used the Dundee Ready Education Environment Measure (DREEM) for academic climate, and the SA-45 (Symptom Assessment-45 Questionnaire was used to assess psychopathological symptomatology.

**Results:**

The mean DREEM global score was low, 95.8 (SD 22.6). Worse perception of the academic climate has been found in females (*t* -2.21, p 0.027), in students of the clinical academic years (t 16.9, *p* < 0.001), and public medical schools ( t 15.6, *p* < 0.001).

The SA45 general index score was high (p90) in 25.6% of participants. In respect of gender, female students presented higher levels of SA45 general index score, depression, interpersonal sensitivity, somatization, anxiety, obsession-compulsion, and phobic anxiety symptoms.

Higher DREEM global and subscale scores corresponded to a higher SA-45 global index score and higher SA-45 subscale scores.

**Conclusions:**

Our study suggests a correlation between a poor perception of academic climate, increased depression, anxiety, and other psychopathological symptoms, with a pattern that varies between different faculties. The perception of academic climate varied between medical schools, as did the psychopathological symptoms scores. Our finding suggests the prevalence of these variables in medical students is, at least in part, attributable to factors directly related to the learning atmosphere.

## Introduction

For several decades, various studies have shown that medical students present poorer mental health than the population of their age, with high levels of stress, and depressive and other psychopathological symptoms [1–5]. This mental health impairment is widespread in many countries, and there appears to be a correlation between poor mental health in medical students, resident doctors, and fully qualified doctors [6, 7].

Multiple factors have been described in the literature as being potentially responsible for the above situation [2, 3]. In the first place, entry to medical courses tends to require high academic qualifications and standards which need to be maintained during training. Slavin [8] identifies various reasons why this problem persists, despite being known about for a long time. These include the conviction that a hard study programme is the best preparation for what is a hard occupation, underestimation of the importance of prevention as opposed to treatment of mental health disorders, a focus in curricular changes on the implementation of new content and teaching methods rather than on the prevention of poor student mental health, the association of unfavourable student mental health conditions with poor institutional quality, and finally an approach that is based more on the level of individual self-care than on the learning environment as a systemic problem.

Other studies have hypothesised about the role of academic climate as a modulator or enhancer of stress, and thus a relevant factor in the development of psychopathological symptoms in medical students [5, 9–11]. Dyrbye [9], in one of the few longitudinal studies that have been conducted, found an association in medical students between an unfavourable perception of the academic environment and higher levels of stress and lower levels of empathy.

The Association of American Medical Colleges includes among its objectives the need, in medical education, to improve “the health and well-being of students” [12]. The educational climate is considered a complex system that depends on the perception of the members of the institution, encompassing aspects that go beyond infrastructure and curriculum, such as interpersonal links, timetables, teaching methods, personal safety, the hidden curriculum, and the organisational culture,—elements that facilitate access to the knowledge necessary to contribute to the optimal management of the teaching–learning process [11–13]. In 1998, the World Federation for Medical Education established that evaluation of the academic environment is one of the fundamental axes in assessments of medical education programmes [14].

The personal and professional development of medical students is strongly influenced by the academic climate in which they spend their learning time. This climate fervently influences their education, their satisfaction with the curriculum, the learning outcomes of the programme and course, and their corresponding professional development [15–17]. Academic climate also appears to be related to well-being and quality of life [18, 19]. Some studies also show associations between academic climate and burnout [9, 18–21], poor quality of sleep and mental health difficulties [22].

The aim of this study is to describe students’ perception of the academic climate in Spanish medical schools and its relationship with psychopathological symptoms. In addition, an assessment is made of the influence of sociodemographic variables of interest, including age, academic year, and type (clinical vs. non-clinical courses), and gender, on the perception held by the students of the educational climate.

## Materials and method

This is an observational, multicentre, cross-sectional study. All medical degree students from the 44 medical schools in Spain, in the second semester of the 2021–22 academic year, were included. New medical schools that have not yet completed all the courses have been excluded.

The instrument used was a self-administered survey developed from a web questionnaire in Google Forms, based on a previously published questionnaire. Demographic variables included were age, gender, academic course (In Spain, a degree in Medicine lasts 6 years), and private vs public schools. The participants, all medical students, were recruited through text messages sent by the Student Delegation Offices in each medical school. They also gave their informed consent before completing the survey. At that time, we estimate the total number of university undergraduates studying medicine in Spain to have been around 42,000.

### Dundee Ready Education Environment Measure (DREEM)

The main variable was *academic climate*, which was measured using the validated Spanish version of the DREEM [22, 23], with a Cronbach’s α reliability coefficient of 0.92. This tool consists of 50 items ranked on a 5-point Likert-type scale. A score is given for each item ranging from 0 (Strongly disagree) to 4 (Strongly agree). The items are classified into 5 subscales:

- Subscale 1: Students’ *perceptions of learning*—Addresses the learner’s view of learning activities and methods, assessing for example whether objectives are clear, learning is student-centred or active learning is promoted. Score 0–48.
- Subscale 2: Students’ *perceptions of teachers*—Addresses the student’s evaluation of the ability and quality of the teachers, their level of knowledge, their communication skills or whether they give correct feedback. Score 0–44.
- Subscale 3: Students’ *academic self-perceptions*—Addresses the learner’s own view of the learning strategies and skills they have developed to solve problems and prepare for their profession. Score 0–32.
- Subscale 4: Students’ *perceptions of atmosphere*—Addresses the quality of the environment in the classroom and in the clinical centres, such as whether it is motivating or offers opportunities to participate or develop relationship skills. Score 0–48.
- Subscale 5: Students’ *social self-perceptions*—Addresses the student’s view of their own social and personal environment in areas such as having assistance in managing stress, having good friendships or the social environment of the educational institution. Score 0–28.

The maximum possible score is 200 points. In accordance with the DREEM User Guide, as well as previous studies, the following relationship is established between the overall results obtained and the quality of the educational climate: 0–50 very poor; 51–100 existence of many problems; 101–150 more positive than negative; 151–200 excellent. The DREEM tool also enables identification of the strengths and weaknesses of a teaching curriculum.

### SA-45 (Symptom Assessment-45 Questionnaire)

The SA45 is a self-report questionnaire of psychopathological symptoms derived from the SCL-90 and developed by Davison et al. [24]. The SA-45 assesses psychological distress in terms of nine primary symptom dimensions and a summary global score. The principal symptom dimensions are labelled somatization, obsessive compulsive, interpersonal sensitivity, depression, anxiety, hostility, phobic anxiety, paranoid ideation, and psychoticism. The global measures are referred to as the Global Severity Index (GSI). Each item is assessed on a Likert-type scale between 0 (“Not at all”) and 4 (“Very much or extremely”). We used the Spanish version validated by Sandín et al. [25] with a Cronbach’s α reliability coefficient of 0.96. The cut-off points were two standard deviations, estimated by Albarado et al. [26].

Descriptive statistics were calculated, and a comparison of means was made using the Student’s t-test, the normality was verified with the test of Komogorov-Smirnov and Levene’s test for homogeneity of variances. Pearson’s correlation coefficient was used for the relationship between quantitative variables. All analyses were carried out bilaterally and with a statistical significance of 0.05 using the JAMOVI 2.2.5 software programme.

### Ethical approval

The study was approved by the Ethics Committee of the University of Murcia (reference 3772/2022) in accordance with the Spanish Medical Research with Human Subjects law. Informed consent was obtained from the study participants. Participation in this anonymous questionnaire was voluntary and participants could refuse to participate or discontinue participation at any time without penalty. The anonymous questionnaires could not be traced back to individuals. Data was stored on secure servers and only people involved in this study had access to it. All methods were performed in accordance with relevant guidelines and regulations.

## Results

A total of 4,374 students from 44 Spanish medical schools participated in the study (response rate 10.17%). Of these, 3,275 were female (74.9%), 1065 male (24.4%) and 34 non-binary (0.8%). Mean age was 21.5 (SD 3.52). Participation by academic year is shown in Table 1.

**Table 1:** Distribution of students by academic year

Academic year	Participants	Age (Mean)	Gender Female /Male	Public/private School
1	855 (19.3%)	19.1	662 / 187	741 / 114
2	782 (17.6%)	20.2	573 / 200	671 / 111
3	867 (19.6%)	21.5	665 / 196	728 / 139
4	781 (17.6%)	22.5	579 / 196	654 /127
5	709 (16.0%)	23.4	520 /186	606 / 103
6	439 (9.9%)	26.0	320 / 115	361 / 78

**Fig. 1:**
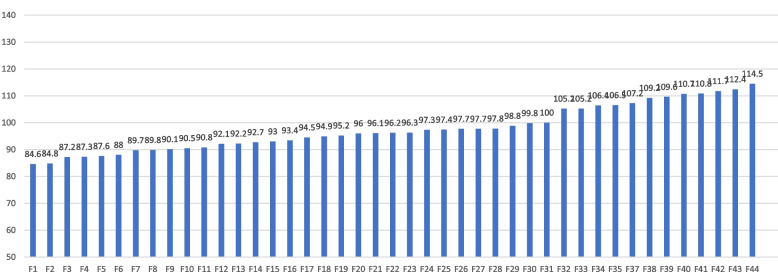
Mean DREEM score in medical schools

The mean DREEM global score was 95.8 (SD 22.6). The lowest score in a medical school was 84.6 and the highest 114.5 (Fig. 1). Only 13 of the 44 medical schools surveyed scored above 100, of which 2 were public and 11 privates. There were no statistically significant differences by region of origin.

The scoring in the different subscales was as follows: Students’ *perception of learning*: Mean 18.9, SD 22.6. (low score, < 24: Teaching is viewed negatively)); Students’ *perceptions of teachers*: Mean 25, SD 5.3 (average score, > 23: Moving in the right direction); Students’ *academic self-perceptions*: Mean 15.7, SD 5.7 (low score, < 16: Many negative aspects); Students’ *perceptions of atmosphere*: Mean 22.5, SD 5.6 (low score, < 22.5: There are many issues that need change); Students’ *social self-perceptions*: Mean 13.8, SD 4.4 (low score, < 14: Not in a nice place).

Significant differences by gender were found in the DREEM global score and in some of the subscales (Table 2). According to the global score, female students have a worse perception of the academic climate (*t* -2.21, p 0.027) than their male peers. This was also the case in the *academic self-perceptions* subscale (*t* -3.764, *p* < 0.001) and in the *perception of learning* subscale (*t* -2.028, p 0.004). No differences were found in the other subscales.

**Table 2:** DREEM score and gender

DREEM Academic climate perception	Female/Male Mean (SD)	95% Confidence Interval	Student’s *t*-test, *p*
Global score	95.5 (22.2) 97.3 (23.5)	94.8–95.9	-2.21, *p* 0.02
Students’ perception of learning	18.9 (5.24) 19.2 (5.49)	15.7–18.7	- 2.028, *p* 0.004
Students’ perceptions of teachers	24.9 (5.69) 25.1 (5.93)	20–24.8	-0.9, ns
Students’ academic self-perceptions	15.5 (5.49) 16.2 (5.75)	15.3–15.9	- 3.764, *p* < 0.001
Students’ perceptions of atmosphere	22.4 (5.19) 22.8 (5.42)	18.8–22.5	-0.9, ns
Students’ social self-perceptions	13.8 (4.32) 14 (4.56)	9.98–13.8	-1.7, ns

**Fig. 2:**
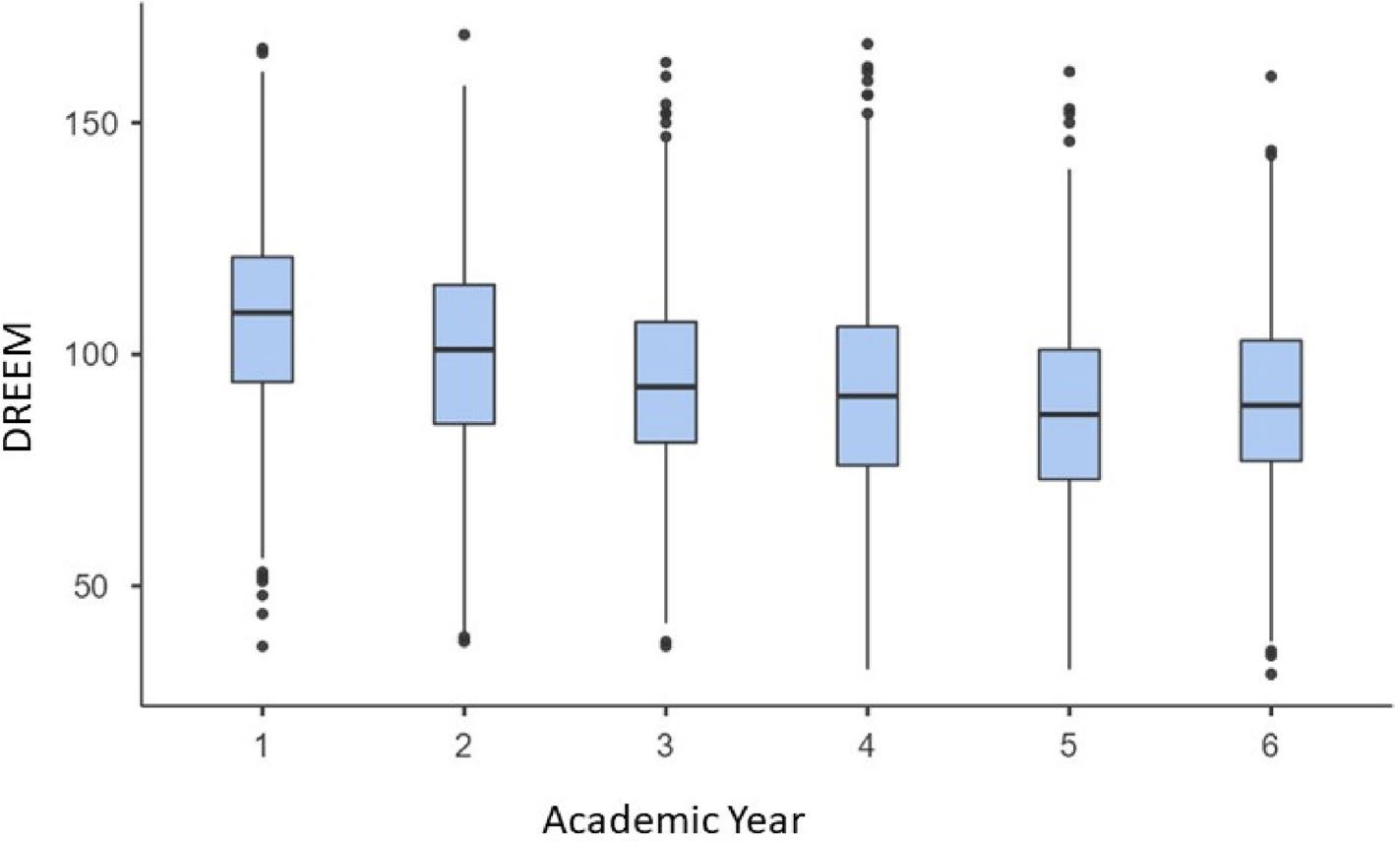
DREEM global score by academic year

In accordance with academic year, students in the final 3 years of the degree (4–6 academic year, clinical course) presented a lower global score in their perception of the academic climate and a lower score in the different subscales (Fig. 2 and Table 3) compared to those in the first academic year (non-clinical course). Table 4 shows the strengths and weaknesses of the programme as identified by the DREEM score.

**Table 3:** DREEM score in preclinical (years 1–3) and clinical (years 4–6) courses

DREEM Academic climate perception	Preclinical/clinical Mean (SD)	95% Confidence Interval	Student’s *t*-test, *p*
Global score	100.7 (21.87) 89.5 (21.89)	90.2–103	16.9, *p* < .001
Students’ perception of learning	19.9 (5.27) 17.6 (5.05)	17.7–20.5	14.7, *p* < .001
Students’ perceptions of teachers	26.0 (5.43) 23.65.89)	23.8–26.4	14.1, *p* < .001
Students’ academic self-perceptions	16.6 (5.41) 14.4 (5.55)	14.5–16.9	13.4, *p* < .001
Students’ perceptions of atmosphere	23.5 (5.12) 21.2 (5.11)	21.3–24.0	15.3, *p* < .001
Students’ social self-perceptions	14.7 (4.42) 12.7 (4.1)	12.8–15.1	15.0, *p* < .001

**Table 4:** Strengths and weaknesses of the program as identified by the DREEM score

**Six best areas of the program identified by high DREEM scores**
DRREM 2: The teachers are experts. Mean score 2.93 (SD 0.85) DREEM 14: I have good friends at this university. Mean score 3.16 (SD 1.08) DREEM 37: The teachers set a good example. Mean score 2.54 (SD 0.89) DREEM 39i: The teachers get angry in class. Mean score 2.55 (SD 1.08) DREEM 46: I live in a pleasant place. Mean score 3.16 (SD 0.99) DREEM 50i: The students annoy the teachers. Mean score 2.64 (SD 1.07)
**Six problem areas of the program identified by low DREEM scores that need to be improved**
DRREM 3: Good support system for students who get stressed out. Mean score 0.67 (SD 0.89) DREEM 4i: I am too tires to enjoy the course. Mean score 1.31 (SD 1.19) DREEM 9i: I am confident I will pass this course. Mean score 1.46 (SD 1.17) DREEM 12: The teaching is student-orientated. Mean score 1.35 (SD 1.18) DREEM 13: I rarely get bored in the classes. Mean score 1.24 (SD 1.07) DREEM 48: The teaching is too teacher-orientated. Mean score 1.07 (SD 1.15)

Statistically significant differences were found between public and private institutions in the perception of the academic climate for the DREEM global score and all its subscales (Table 5).

**Table 5:** DREEM score in Public University and Private University

Academic climate (DREEM)	Public University / Private University mean (SD)	95% Confidence Interval	Student’s *t*-test, p
Global score	Public 93.7 (21.7) Private 108 ( 23.3)	93–106	-15.6, *p* < .001
Students’ perception of learning	Public 18.5 (5.1) Private 21.2 (5.6)	18.3–20.8	-12.5, *p* < .001
Students’ perceptions of teachers	Public 24.5 (5.7) Private 27.5 (5.5)	24.3–27.1	-12.8, *p* < .001
Students’ academic self-perceptions	Public 15.2 (5.4) Private 18.5 (5.6)	15–18	-14.4, *p* < .001
Students’ perceptions of atmosphere	Public 22.1 (5.1) Private 24.7 (5.4)	21.9–24.3	-12.0, *p* < .001
Students’ social self-perceptions	Public 13.4 (4.2) Private 16.1 (4.8)	13.3–15.8	-15.1, *p* < .001

The SA45 general index score was high (p90) in 25.6% of participants. In respect of gender, female students presented higher levels of SA45 general index score, depression, interpersonal sensitivity, somatization, anxiety, obsession-compulsion, and phobic anxiety symptoms (Table 6).

**Table 6:** SA45 score and gender

	**Female/Male** **Mean (SD)**	**t student; *p***
SA-45 global index score	51.1 (31.8) 43.1 (29)	7.28; < .001
Drepression	9.1 (5.6) 7.98 (5.5)	5.95; < .001
Hostility	2.5 (3.5) 2.65 (3.6)	-0.56; 0.576
Interpersonal sensitivity	7.25 (5.1) 6.04 (4.8)	6.81; < .001
Somatization	5.6 (5.1) 3.73 (4.4)	10.84; < .001
Anxiety	8.09 (5.06) 6.31 (4.9)	10.19; < .001
Psychoticism	1.93 (2.8) 1.98 (2.8)	-0.55; 0.580
Obsession-compulsion	8.11 (4.9) 7.16 (4.8)	5.53; < .001
Phobic anxiety	3.47 (3.9) 2.52 (3.3)	7.26; < .001
Paranoid ideation	4.87 (4.02) 4.72 (3.8)	1.10; 0.271

In almost all cases, higher DREEM global and subscale scores corresponded to a higher SA-45 global index score and higher SA-45 subscale scores (Table 7).

**Table 7:** Correlation between perception of academic climate and psychopathological symptoms

Academic climate (DREEM)	SA-45 global index score Pearson, *p*	Depression Pearson, *p*	Anxiety Pearson, *p*	Hostility Pearson, *p*	Interpersonal sensitivity Pearson, *p*	Somatization Pearson, *p*	Psychoticism Pearson, *p*	Obsession-compulsion Pearson, *p*	Phobic anxiety Pearson, *p*	Paranoid Ideation Pearson, *p*
Global score	*P* -0.334, p < 0.001	*P* -0.437, *p* < 0.001	*P* -0.334, *p* < 0.001	*P* -0.218, *p* < 0.001	*P* – 0.3, *p* < 0.001	*P* -0.22, *p* < 0.001	*P* – 0.090, *p* < 0.001	*P* -0.265, *p* < 0.001	*P* -0.208, *p* < 0.001	*P* -0.180, *p* < 0.001
Students’ perception of learning	*P* -0.190, *p* < 0.001	*P* -0.296, *p* < 0.001	*P* -0.200, *p* < 0.001	*P* -0.115, *p* < 0.001	*P* – 0.175, *p* < 0.001	*P* -0.115, *p* < 0.001	*P* -0.073 *p* 0.002	*P* -0.181, *p* < 0.001	*P* -0.092, *p* < 0.001	*P* -0.067, *p* < 0.001
Students’ perceptions of teachers	*P* -0.302, *p* < 0.001	*P* -0.348, *p* < 0.001	*P* -0.283, *p* < 0.001	*P* 0.220, *p* < 0.001	*P* -0.276, *p* < 0.001	*P* -0.208, *p* < 0.001	*P* -0.109, *p* < 0.001	*P* -0.212, *p* < 0.001	*P* -0.213, *p* < 0.001	*P* -0.209, *p* < 0.001
Students’ academic self-perceptions	*P* -0.372, *p* < 0.001	*P* -0.390, *p* < 0.001	*P* -0.366, *p* < 0.001	*P* -0.226, *p* < 0.001	*P* -0.334, *p* < 0.001	*P* -0.235, *p* < 0.001	*P* -0.127, *p* < 0.001	*P* -0.319, *p* < 0.001	*P* -0.239, *p* < 0.001	*P* -0.184, *p* < 0.001
Students’ perceptions of atmosphere	*P* -0.231, *p* < 0.001	*P* -0.303, *p* < 0.001	*P* -0.254, *p* < 0.001	*P* -0.154, *p* < 0.001	*P* -0.205, *p* < 0.001	*P* -0.165, *p* < 0.001	*P* – 0.05, *p* < 0.001	*P* -0.160, *p* < 0.001	*P* -0.138, *p* < 0.001	*P* -120, *p* < 0.001
Students’ social self-perceptions	*P* -0.340, *p* < 0.001	*P* -0.447, *p* < 0.001	*P* -0.338, *p* < 0.001	*P* -0.221, *p* < 0.001	*P* – 0.3, *p* < 0.001	*P* -0.221, *p* < 0.001	*P* – 0.099, *p* < 0.001	*P* -0.271, *p* < 0.001	*P* -0.207, *p* < 0.001	*P* -0.195, *p* < 0.001

## Discussion

The role played by the academic climate in course development is well established, but there is growing interest in its implications for mental health. The mental health of medical students is a matter of concern and, given the impact it has, and the numbers involved, it is crucial to determine its causes and have improvement strategies available. The present study, conducted in 2022 in all medical schools in Spain, analyses the academic climate and student psychopathological symptoms in the first study of this scope on a national scale assessing these variables. Moreover, the sample (4,433 participants) is large enough to endow the data presented here with reliability, thereby enabling conclusions to be drawn.

A mean overall score of 95.8/200 was found for the DREEM items. According to the practical guide of McAleer and Roff [27], a mean score between 50 and 100 indicates potential problems. Our results contrast with those reported in previously published studies in that the scores obtained in most medical schools tend to range between 100 and 120, with the highest often found in European-based schools (UK, German, French) [28–30], whereas scores below 100 are found in medical faculties in non-western countries [31, 32], as Iran or Saudi Arabia. In a study conducted in 5 Spanish medical schools [13], the mean score was 116.2 in the second year of the course and 104.8 in the fourth year.

The low scores found in almost all the DREEM subscales suggest the following [27]: in the students’ perceptions of learning that the *teaching is viewed negatively*; in their academic self-perceptions that there are *many negative aspects*; in their perceptions of atmosphere that there *are many issues that need change*; and in social self-perceptions that they are *not in a nice place*. Only in student’s perceptions of teachers was there found to be a positive trend, with the result suggesting that the school is *moving in the right direction*. This result is in agreement with the fact that 4 of the 6 best items are correlated to the teaching staff (Table 4).

These low numbers could be due to the influence of the COVID pandemic, but in Spain different Autonomous Communities implemented different university lockdown measures, and no differences were found in DREEM between these communities. Some preliminary studies on the influence of the pandemic show an impact on mental health, but little influence on the academic climate [30], although one study did show a greater impact on the academic climate [33]. More studies are required to establish the causes of the particularly low scores in Spanish medical schools.

The scores regarding the perception of the academic climate were lower among female students and as the students advanced in the course. Higher academic climate scores were recorded in private institutions rather than in the public ones, as in other previous studies [13, 34]. It is necessary to pass a general national exam to access medical school in Spain. A very high qualification is necessary to access public medical schools. Each of the private schools has specific admission systems.

The results obtained in terms of psychopathological symptoms were similar to those obtained in previous studies, both at national and international scale [1–6, 30, 35–38]. Female students presented indications of poorer mental health and greater psychopathological symptoms, with both variables increasing generally as the course progresses, as also reported in other previous studies [30, 36–38].

In our study, perception of academic climate showed a high correlation with the level of psychopathological symptoms. Students who perceived the academic climate as less favourable were more likely to develop higher levels of anxiety, depression, and other psychopathological symptoms. It should also be noted that the item with the lowest score on the DREEM scale (0.67/4) was “There is a good support system for students who become stressed”.

The perception of academic climate varied between medical schools, as did the psychopathological symptoms scores. This finding suggests the prevalence of these variables in medical students is, at least in part, attributable to factors directly related to the learning atmosphere, as also indicated in other studies [8, 9, 21, 38].

Multiple programmes have approached the problem of medical students’ mental health as an individual but not as a global problem. As in medical poor mental health analyses, “a system-level problem requires system-level responses” [39]. Wasson et al. [18] identified, in a systematic review, 28 interventions designed to promote the well-being of medical students. The authors found evidence, albeit limited, suggesting that some specific academic climate interventions were associated with improved emotional well-being among medical students. However, the authors acknowledged that higher quality studies are needed to corroborate this claim.

The aim of medical schools is to produce trained, competent, and professional doctors equipped to care for the sick, advance the science of medicine and promote public health [2]. However, the epidemic of poor mental health in medical students implies a failure in this goal. There is a clear gap between what is known and what is done, with medical schools continuing to focus more on the acquisition of knowledge and skills than on the holistic training of doctors [40]. Slavin [8] makes the point that “medical schools must step forward to address the mental health crisis among medical students, and solutions cannot come from the mental health side alone; the problem must be seen as an environmental health problem”.

The great challenge would be to promote self-care strategies and approach to stress and poor mental health. It would be important that mental health and prevention of stress, depressions or anxiety had preventive programs general, as well as specific programs of detection and approach to high-risk students.

This study has some limitations. The nationwide nature of the study design is a factor that speaks to the generalizability of our results. However, although the number of responses was high, not all the results will be applicable to each university, since the response rate varied widely. The use of a self-administered tool rather than structured clinical interviews may also be a limitation because, although used in many studies, the scoring of psychopathological symptoms may not accurately reflect the severity of a mental health problem. It is also possible that students who were especially sensitive to these issues or were aware of having these problems were more predisposed to complete the survey than others. Finally, the sampling procedure was non probabilistic since participation was offered to everyone but only those who wanted to participate.

In conclusion, our study suggests a high correlation between a poor perception of academic climate and increased psychopathological symptoms, with a pattern that varies between different faculties. Further research is needed to detect the specific modifiable aspects of the learning environment that are most strongly related to poor mental health, as well as to determine the best strategies to create and maintain a learning environment that ensures the correct professional development of medical students.

## Data Availability

The datasets used and/or analysed during the current study are available at http://hdl.handle.net/10201/128383.
